# 

**Published:** 1906-07-28

**Authors:** 


CONTENTS.

**The Medical Inspection of Scholars** - 291
**A Plea for the Eye** - 292
**Annotations** - 293
**Medical Opinion and Movement** - 294
**Graduate Teaching Facilities in London Hospitals** - 295
**Hospital Clinics—**
Some Remarks on the Diagnosis and Complications of Diphtheria - 297
Leucocytosis after Operations - 299
**Special Analytical and Health Commission on Tinned and Preserved Food** - 300
**Practical Notes on Diagnosis and Treatment** - 301
**The Book World of Medicine and Science** - 302
**Hospital Administration—**
Current Hospital Topics - 303
New Sanitary Wing at the Boston Hospital - 304
Social and Poor-law Problems - 304
Structural and Practical Departments - 305
**Editor's Letter-Box** - 305

*NURSING SECTION.*

**Notes on News from the Nursing World—**
The Queen and the Jubilee Institute—Honour for Miss Hughes—Nursing in India and Lady Curzon—Why the Army Nursing Service is Attacked—Mr. Haldane on the Mobilisation of Nurses—Enrolment in Time of War—Pleasing Incident at the London Temperance Hospital—Poor-law Superannuation—An American Nurse and her Patient—Cubicles in the Basement—Royal Visit to Whitechapel Infirmary—Examinations in Australia—The Annual Outing to Guy's Nurses—Garden Fête at Stamford Infirmary—A Strawberry Tea—The Offices of the Pension Fund—Nurses and Tennis—The Garden as an Isolation Hospital—The Little Hoppers' Hospital—Short Items - 239-242
**The Nursing Outlook** - 243
**Abdominal Surgery** - 244
**The Nurse's Clinic** - 245
**Midwifery Amongst the Poor** - 246
**Nursing Incurables** - 247
**Incidents in a Male Nurse's Life** - 248
**Practical Hints** - 248
**Presentations** - 249
**Should Nurses be Registered?** - 249
**The Nurses' Bookshelf** - 250
**Everybody's Opinion** - 251
**Appointments** - 251
**Novelties for Nurses** - 251
**Notes and Queries** - 252
**For Reading to the Sick** - 252

